# Plasma Zonulin Levels in Childhood Nephrotic Syndrome

**DOI:** 10.3389/fped.2019.00197

**Published:** 2019-05-16

**Authors:** Howard Trachtman, Debbie S. Gipson, Kevin V. Lemley, Jonathan P. Troost, Christian Faul, Debra J. Morrison, Suzanne M. Vento, Dong-hyun Ahn, Judith D. Goldberg

**Affiliations:** ^1^Division of Nephrology, Department of Pediatrics, NYU School of Medicine, New York, NY, United States; ^2^Division of Nephrology, Department of Pediatrics, University of Michigan, Ann Arbor, MI, United States; ^3^Division of Nephrology, Department of Pediatrics, Children's Hospital Los Angeles, Keck School of Medicine, University of Southern California, Los Angeles, CA, United States; ^4^Division of Nephrology, Department of Medicine, University of Alabama at Birmingham, Birmingham, AL, United States; ^5^Perlmutter Cancer Center, New York, NY, United States; ^6^Department of Population Health, NYU School of Medicine, New York, NY, United States

**Keywords:** nephrotic syndrome, minimal change disease, focal segmental glomerulosclerosis, zonulin, PAR-2

## Abstract

**Objective:** We conducted this study to test the hypothesis that plasma zonulin levels are elevated in pediatric patients with nephrotic syndrome compared to healthy controls.

**Study Design:** Plasma zonulin levels were measured by ELISA in 114 children enrolled in the NEPTUNE study. Clinical and laboratory data were retrieved from the NEPTUNE database.

**Results:** The median age of the patients was 10 (IQR = 5 to 14) years, 59 were male, 64 had minimal change disease, 47 focal segmental glomerulosclerosis, median eGFR was 96 (IQR = 80 to 114) ml/min/1.73 m^2^, and median urine protein:creatinine ratio was 0.5 (IQR = 0.1 to 3.4) (g:g). The plasma zonulin level was 14.2 ± 5.0 vs. 10.2 ± 2.5 ng/ml in healthy adults in a report using the same assay kit, *P* = 0.0025. These findings were confirmed in an independent cohort of children with nephrotic syndrome compared to healthy age-matched controls, *P* = 0.01. Zonulin concentrations did not differ in children with minimal change disease vs. focal segmental glomerulosclerosis, frequently relapsing vs. steroid-dependent vs. steroid-resistant clinical course, and were not influenced by the immunosuppressive treatment regimen. There was no relationship between plasma zonulin levels and the absolute or percentage change in proteinuria from enrollment until the time of the zonulin assay.

**Conclusion:** Plasma zonulin levels are elevated in childhood nephrotic syndrome regardless of level of proteinuria or specific treatment. The cause of the high plasma zonulin levels and whether zonulin contributes to glomerular injury requires further study.

## Background

Primary nephrotic syndrome (NS) in childhood is a rare condition with an annual incidence of 2–7/100,000 (1). It is most commonly caused by either minimal change disease (MCD) or focal segmental glomerulosclerosis (FSGS), which are distinguished histologically and clinically by responsiveness to treatment. The first line therapy for children with NS is corticosteroids. Other immunosuppressive agents are used in patients who are resistant to corticosteroids or who develop serious side effects (2). Patients with MCD consistently achieve remission of proteinuria when treated with corticosteroids or other immunosuppressive medications and have an excellent long-term prognosis with resolution of the disease and preservation of kidney function. In contrast, nearly 50% children with FSGS are resistant to currently available therapies and are at high risk of progression to end stage kidney disease. MCD likely results from a disturbance in the immune system. This is based on recent genetic studies linking steroid responsive NS to polymorphisms in the HLA-DQ and DR locus (3, 4). In addition, the therapeutic response to corticosteroids and agents that target T and B cells lends further support for a role of the immune system in the pathogenesis of MCD (5). FSGS is more heterogeneous and may reflect the presence of a circulating permeability factor, a genetic abnormality in the podocyte that leads to proteinuria, or other environmental or infectious etiologies (6, 7). For both disease entities, there is a pressing need to identify new approaches to treatment that are safe and effective.

Case reports suggest that some children with NS are responsive to dietary modifications (8). Lagrue reported two case series in which, in the aggregate, more than half of children with NS had a favorable response to a variety of dietary interventions (9, 10). Two out of 3 children placed on a gluten-free diet (GFD) had a complete remission. Uy et al. (11) outlined the potential utility of gluten- and dairy-free diets in childhood NS. In a recent retrospective study, 8 children with difficult-to-manage NS benefited from placed on a GFD for 3.4 ± 4.3 years (range, 0.6–14 years). However, the literature detailing the response of patients with NS to dietary interventions is limited and fairly old. In addition, there is no description of renal histology or precise delineation of the disease pattern. In a recent retrospective study, there was a clinical benefit with a ≥50% reduction in annual relapses rate in 7 children and a decreased number and dosage of immunosuppressive medications prescribed in all cases (12).

In celiac disease, zonulin is released from enterocytes after exposure to gliadin, activates protease-activated receptor 2 (PAR2) in a paracrine manner, and perturbs the actin cytoskeleton and cell-cell junctions in the gut epithelium (13–16). PAR2 is expressed on podocytes and a gluten-induced elevation in the serum zonulin level may increase binding of the ligand to the receptor. This in turn may modulate the interaction between PAR2 and PAR3, which has been implicated in cell signaling and podocyte structure and function (17–20). Alterations in the podocyte cytoskeleton influence cell motility and attachment to the glomerular basement membrane leading to proteinuria (21). Furthermore, a zonulin knock-in mouse model with elevated serum zonulin levels develops albuminuria (unpublished data). Based on these clinical and pre-clinical findings, we conducted this study to test the hypothesis that plasma zonulin levels are elevated in pediatric patients with NS compared to published estimates from healthy controls.

## Methods

### Patients

The eligibility criteria and the clinical procedures of the NEPTUNE multicenter, observational cohort study have been published previously (see Appendix) (22). In brief, NEPTUNE, which is part of the National Institutes of Health (NIH) Rare Disease Clinical Research Network (RDCRN), has enrolled adults and children with MCD, FSGS, and membranous nephropathy at the time of clinically indicated renal biopsy. Initial visits include an extensive clinical history, physical examination, collection of urine, blood and renal tissue samples, and assessments of quality of life and patient-reported outcomes. Follow-up history, physical measures, urine and blood samples, and questionnaires are obtained every 4 months in the first year and biannually, thereafter. Samples (urine, serum, plasma, and DNA) are stored in a biorepository and available for use in approved ancillary studies.

The following clinical and laboratory data were collected in accord with the approved NEPTUNE ancillary study protocol: age, gender, race/ethnicity, disease group, treatment, blood pressure (BP), estimated glomerular filtration rate (eGFR), quantitative proteinuria, and serum albumin and cholesterol concentrations.

A validation cohort of children with NS treated at NYU Langone Health who were not enrolled in NEPTUNE was recruited for measurement of plasma zonulin level concurrently with samples collected from healthy age-matched control children who were enrolled in an independent NIH sponsored observational cohort study, C-PROBE. The C-PROBE cohort study is an observational cohort and biobanking study of patients with chronic kidney disease and healthy controls (23).

### Samples and Analytical Methods

Plasma specimens collected from all patients ≤ 18 year old with MCD or FSGS enrolled in the NEPTUNE study were tested for plasma zonulin concentration. Most of the samples were available for testing from the 4 (*n* = 86) or 8 month (*n* = 19) protocol visit with the remaining 9 samples collected at the 12–30 month visits. The selection of the visit for which a sample was retrieved for zonulin measurement was determined by availability of specimens in the NEPTUNE biorepository. Clinical and laboratory data were retrieved coincident with the visit when the zonulin level was measured. Plasma zonulin levels were measured in the NYU Core Laboratory using a commercially available ELISA kit (Immunodiagnostik AG).

### Statistical Methods

Descriptive summaries are provided for the final sample of subjects with zonulin measurements. Continuous data are summarized as medians and interquartile ranges and with boxplots to display the distributions. Categorical data are summarized as frequencies and percentages. Associations between Zonulin levels and continuous variables, such as proteinuria and eGFR, were evaluated using Spearman non-parametric correlation coefficients. Zonulin levels were compared across levels of categorical variables, such as diagnosis, using Kruskal-Wallis non-parametric analysis of variance tests. Zonulin levels were also compared to the healthy reference population values with mean = 10.2 and SD = 2.5 using a one-sample *t*-test (24). *P*-values < 0.05, 2-sided were considered statistically significant. No adjustments were incorporated for multiple testing. All analyses were completed using SAS V9.4 and R.

## Results

The clinical and laboratory features of the study patients at baseline are summarized in Table 1. The median age was 10 (IQR = 5–14) years old and the majority of patients were male. The distribution of disease activity was almost evenly divided between infrequently relapsing, frequently relapsing/steroid dependent, and steroid resistant. Sixty-four children had MCD and 47 had FSGS. The median blood pressure and level of kidney function were normal. By design, urinary protein excretion was elevated at enrollment into NEPTUNE. Clinically significant nephrotic-range proteinuria (urine protein:creatinine ratio >2 g:g) and hypercholesterolemia (cholesterol concentration >170 mg/dl) were present in nearly a third of patients at baseline.

**Table 1:** Clinical and laboratory features of the study sample by elevated zonulin level (*n* = 114).

	**All Patients**	**Normal Zonulin (≤15.2 ng/ml)**	**Elevated Zonulin (>15.2ng/ml)**	***p***
	***n* = 114**	***n* = 60**	***n* = 54**	
**Age (year), median (IQR)**	10 (5, 14)	11 (5, 14)	9 (5, 14)	0.72
0–6, n (%)	2 (2)	2 (3)	0 (0)	
7–12, n (%)	41 (36)	20 (33)	21 (39)	
13–17, n (%)	32 (28)	15 (25)	17 (31)	
Unknown, n (%)	39 (34)	23 (38)	16 (30)	
**Gender**				0.05
Male, n (%)	59 (52)	36 (60)	23 (43)	
Female, n (%)	53 (46)	22 (27)	31 (57)	
Unknown, n (%)	2 (2)	2 (3)	0 (0)	
**Race**				0.18
Multi-racial, n (%)	9 (8)	4 (7)	5 (9)	
Asian/Asian American, n (%)	10 (9)	3 (5)	7 (13)	
Black/African American, n (%)	44 (39)	20 (33)	24 (44)	
White/Caucasian, n (%)	45 (39)	29 (48)	16 (30)	
Unknown, n (%)	6 (6)	4 (7)	2 (4)	
**Diagnosis**				0.41
MCD, n (%)	64 (56)	32 (53)	32 (59)	
FSGS, n (%)	47 (41)	25 (42)	22 (41)	
Non-biopsied, n (%)	2 (1)	2 (3)	0 (0)	
Other, n (%)	1 (1)	1 (2)	0 (0)	
**Clinical disease activity**				0.57
IRNS, n (%)	27 (24)	12 (20)	15 (28)	
FRNS/SDNS, n (%)	41 (36)	25 (42)	16 (30)	
SRNS, n (%)	30 (26)	15 (25)	15 (28)	
Unknown, n (%)	16 (14)	8 (13)	8 (15)	
SBP %tile, Median (IQR)	71 (46, 91)	71 (47, 95)	69 (42, 89)	0.65
DBP %tile, Median (IQR)	73 (43, 88)	70 (39, 87)	73 (46, 89)	0.63
Urine protein: creatinine ratio (g/g) at screening, Mean (SD)	4.8 (1.8, 9.6)	4.9 (2.2, 10.6)	4.6 (1.2, 9.2)	0.55
Urine protein: creatinine ratio(g/g) at baseline visit, Median (IQR)	1.4 (0.2, 5.3)	2.7 (0.3, 5.7)	0.8 (0.1, 4.7)	0.11
Urine protein: creatinine ratio(g/g) at visit with zonulin measurement, Median (IQR)	0.5 (0.1, 3.4)	0.7 (0.2, 5.5)	0.4 (0.1, 1.4)	0.04
eGFR (mL/min/1.73 m^2^) at zonulin assessment, Median (IQR)	96 (80, 114)	96 (76, 115)	97 (80, 113)	0.98
Serum albumin (mg/dL) at zonulin assessment, Median (IQR)	3.5 (2.7, 4.2)	3.2 (2.4, 4.0)	3.8 (3.2, 4.3)	0.06
Cholesterol (mg/dL) at zonulin assessment, Median (IQR)	198 (163, 289)	216 (166, 339)	192 (162, 261)	0.30

The mean plasma zonulin level in the total cohort of children with NS was significantly elevated compared to healthy adults in whom the circulating concentration was measured with the same assay kit (24). There are few published data on plasma zonulin levels in healthy children and adolescents but the mean concentration in the NEPTUNE participants exceeded the value in the limited sample of children of comparable age (25). Of note, 27% of the patients with NS had a plasma zonulin concentration above 17.5 ng/ml, >3 SD above the mean value in adult controls. To validate the findings in the NEPTUNE participants, we measured plasma zonulin levels in an independent cohort of children with NS treated at NYU Langone Health (*n* = 18) and compared them to a group of healthy age-matched children (*n* = 15) who were enrolled in the CPROBE cohort study. In the controls, the median age was 14 (IQR = 12 to 17), 9 were female, 3 were Hispanic ethnicity, and 4 were Black/African American, 10 White/Caucasian and 1 chose not to report race. The plasma zonulin levels were 18.6 ± 4.2 vs. 15.2 ± 2.7 ng/ml in the children with NS and healthy controls, respectively, *P* < 0.02. The difference is comparable to that observed in the NEPTUNE patients and the values reported in the literature for healthy children.

**Figure 1:**
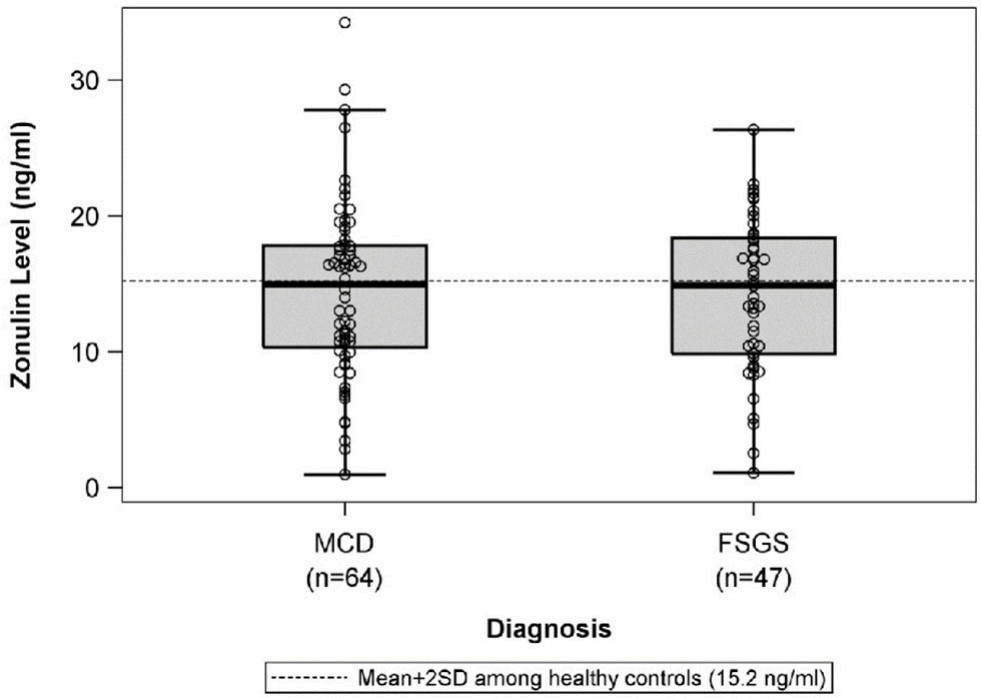
The box plots illustrate the mean, 25th and 75th percentiles, and 2SD boundaries of the plasma zonulin levels in children with MCD (*n* = 64) vs. FSGS (47). The dashed line indicates-the mean+2SD plasma zonulin level in healthy adult control participants. One sample *t*-test of MCD vs. healthy controls (mean = 10.2; SD = 2.5): *p* < 0.001. One sample *t*-test of FSGS vs. healthy controls (mean = 10.2; SD = 2.5): *p* < 0.001. SD, Standard deviation; MCD, minimal change disease; FSGS, focal segmental glomerulosclerosis.

**Figure 2:**
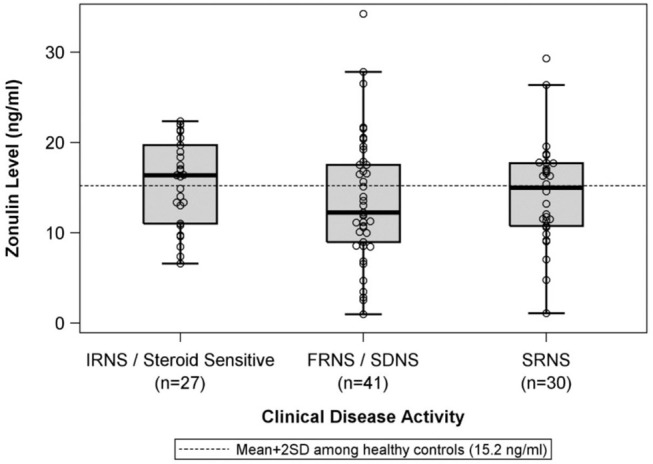
The box plots illustrate the mean, 25th and 75th percentiles, and 2SD boundaries of the plasma zonulin levels in children with IRNS/SSNS (*n* = 27) vs. FRNS/SDNS (*n* = 41) vs. SRNS (*n* = 30). The dashed line indicates-the mean+2SD plasma zonulin level in healthy adult control participants. One sample *t*-test of IRNS/Steroid sensitive vs. healthy controls (mean = 10.2; SD = 2.5): *p* < 0.001. One sample *t*-test of FRNS/SDNS vs. healthy controls (mean = 10.2; SD = 2.5): *p* < 0.001. One sample *t*-test of SRNS vs. healthy controls (mean = 10.2; SD = 2.5): *p* < 0.001. SD, Standard deviation; IRNS, infrequently relapsing nephrotic syndrome; FRNS, frequently relapsing nephrotic syndrome; SDNS, steroid dependent nephrotic syndrome; SRNS, steroid resistant nephrotic syndrome.

**Figure 3:**
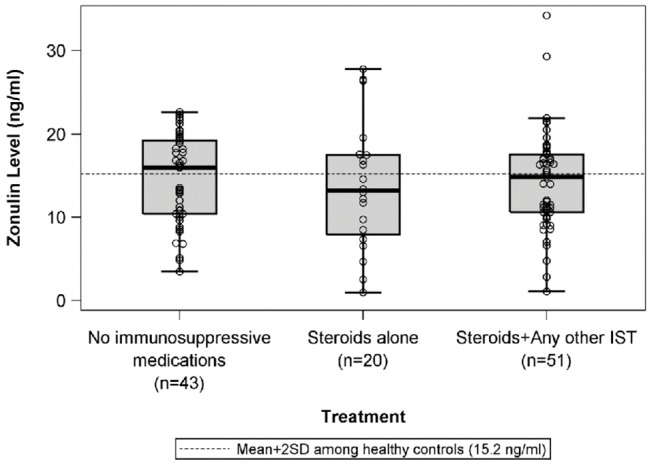
The box plots illustrate the mean, 25th and 75th percentiles, and 2SD boundaries of the plasma zonulin levels in children on no immunosuppressive medications (*n* = 43) vs. steroids alone (*n* = 20) vs. Steroids plus another immunosuppressive medication (*n* = 51) The dashed line indicates-the mean+2SD plasma zonulin level in healthy adult control participants. One sample *t*-test of No immunosuppressive medications vs. healthy controls (mean = 10.2; SD = 2.5): *p* < 0.001. One sample *t*-test of Steroids alone vs. healthy controls (mean = 10.2; SD = 2.5): *p* = 0.06. One sample *t*-test of Steroids + Any other IST vs. healthy controls (mean = 10.2; SD = 2.5): *p* < 0.001. SD, Standard deviation; IST, immunosuppressive therapy.

There were no differences in mean plasma zonulin levels in patients with MCD 14.3 vs. FSGS 14.2 ng/ml (Figure 1). Moreover, there was no difference in plasma zonulin level when the patients were categorized by clinical disease activity—frequently relapsing/steroid dependent, infrequently relapse, and steroid resistant (Figure 2). Forty-eight percent of children were on immunosuppressive treatment at enrollment and this percentage increased to 67% at the time of sample collection for zonulin determination. The plasma zonulin levels did not differ in those not receiving immunosuppressive medication, on corticosteroids alone, or on corticosteroids plus another immunosuppressive medication (Figure 3).

**Figure 4:**
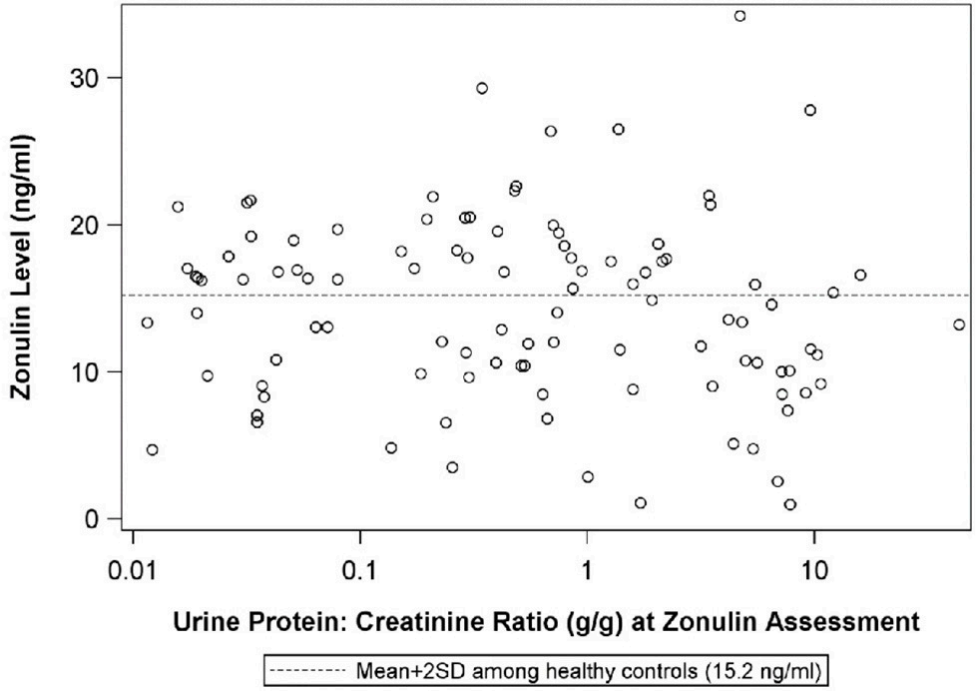
The scatterplot illustrates the relationship between plasma zonulin level and the urine protein:creatinine ratio at the time of the zonulin measurement. The dashed line indicates-the mean+2SD plasma zonulin level in healthy adult control participants. Spearman's Rho = −0.10, *p* = 0.33, R^2^ = 0.01. SD, Standard deviation.

**Figure 5:**
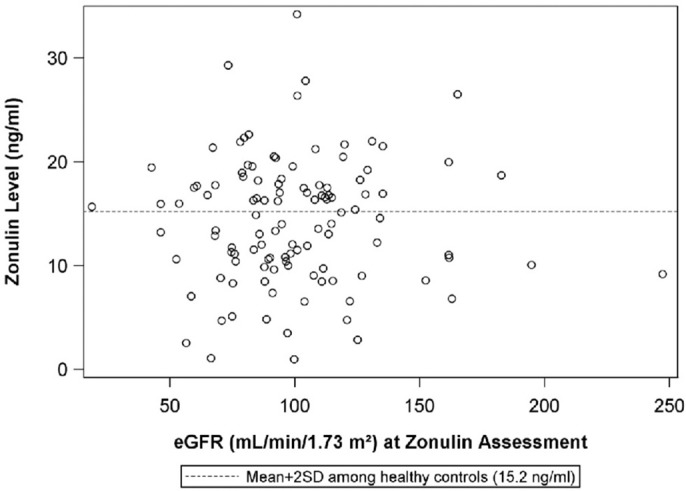
The scatterplot illustrates the relationship between plasma zonulin level and the urine protein:creatinine ratio at the time of the zonulin measurement. The dashed line indicates-the mean+2SD plasma zonulin level in healthy adult control participants. Rho = 0.083 *p* = 0.76, R^2^ < 0.01. eGFR, estimated glomerular filtration rate; SD, Standard deviation.

The median urine protein:creatinine ratio at the visit when plasma zonulin was 0.5 (IQR: 0.1 to 3.4}. There was no correlation between plasma zonulin concentration and the level at proteinuria at baseline or the next two visits including the visit when the plasma zonulin level was measured (Figure 4) nor between zonulin and eGFR on the day of the visit when the plasma zonulin level was determined (Figure 5). There was no association between plasma zonulin levels and absolute or percentage change in proteinuria (data not shown). In an effort to determine if there was a relationship between plasma zonulin levels and the time to the next remission, we examined 61 patients who were not in a complete remission and who had had subsequent follow-up data. In this subgroup of patients, the median follow-up was 41 months (IQR: 28 to 49). There were 37 patients who subsequently reached a complete remission and the median time to achieve remission was 21 months. Zonulin was not associated with time to remission (*P* = 0.27), regardless of whether the analysis was performed based on untransformed, transformed, or categorized plasma zonulin levels.

When the patients were divided into two groups, one with nephrotic-range proteinuria and the other with sub-nephrotic range proteinuria, there was a trend toward a higher plasma zonulin level in the latter group (urine protein: creatinine ratio < 2 g:g, *n* = 72) whose median zonulin level was 16.3 ng/ml [interquartile range = 10.5–18.9] compared to the former group of 31 patients with urine protein: creatinine ratio ≥2 g:g whose median zonulin concentration was 11.6 ng/ml [interquartile range = 9.0–16.6] (*P* = 0.08). The mean plasma zonulin level in both subgroups was higher than the concentration reported in healthy controls and the mean values were more similar between subgroups (mean of 14.8 in urine protein:creatinine ratio ≥2 g:g vs. mean of 13.1 in urine protein:creatinine ratio < 2 g:g vs. mean of 10.2 in healthy controls).

Although there was no correlation between plasma zonulin level and blood pressure or serum cholesterol level, there was a direct relationship with the serum albumin level (*r* = 0.24, *P* = 0.04). Among most routinely available laboratory tests (proteinuria, eGFR or cholesterol concentration), there were no significant predictors of an elevated plasma zonulin level.

## Discussion

In this study, we demonstrate that plasma zonulin levels are elevated in pediatric patients with NS regardless of level of proteinuria or specific therapy. This finding, based on analysis of samples collected in patients enrolled in NEPTUNE was confirmed in a validation cohort of children with NS in whom plasma zonulin level was measured concurrently with a group of healthy age-matched pediatric controls. Plasma zonulin levels correlate with serum albumin level and are numerically higher in those with sub-nephrotic range vs. nephrotic-range proteinuria. There were no clinical or laboratory values that were significant predictors of the plasma zonulin concentration. In a recent study by Lukaszyk et al. (26), plasma zonulin levels were lower in patients with early stage CKD compared to healthy adults, a finding that may account for the lower plasma levels in the children with nephrotic vs. sub-nephrotic proteinuria in the NEPTUNE cohort. The cause of the high plasma zonulin level in children with NS and how it may act on the podocyte to increase proteinuria requires further study.

There are no established normative values for plasma zonulin levels in children and most studies, like ours, compare the concentration in a target population with the level in healthy controls. Plasma zonulin levels, generally measured with the commercial ELISA kit used in this study, have been demonstrated to be higher in patients with celiac disease (27), sepsis (28), and polycystic ovary syndrome (29) but similar in children with autism compared to age-matched healthy controls (25). The latter study showed an inverse relationship between age and plasma zonulin levels. However, the plasma zonulin levels documented in the NEPTUNE cohort of children with NS exceeded the mean circulating concentration measured in all of these prior studies including children of comparable age in the autism study (25). Two studies have reported substantially higher plasma zonulin levels in healthy adults and those with obesity, hyperlipidemia and various gastrointestinal disorders excluding celiac disease compared to the other reports and the current patient series (26, 30). This discrepancy may relate to different procedures for sample collection and/or processing and the inherent variability in plasma zonulin levels based on a single determination (27).

The strength of this study is the fully phenotyped cohort. The NEPTUNE study represents one of the largest well-characterized groups of pediatric patients with NS secondary to MCD or FSGS (21). Nonetheless, we acknowledge that sample size may still be limited to detect differences in zonulin concentration in patient subgroups based on specific clinical features or response to treatment(s). True baseline samples prior to treatment were not routinely available. In addition, samples were retrieved for zonulin assay at different time intervals from baseline. The vast majority of the specimens were collected at 4 (75%) and 8 (17%) months with only 8% obtained at later time points. The NEPTUNE protocol did not mandate therapy. Patients received usual clinical management prescribed by their clinical nephrologist during NEPTUNE participation and sample acquisition. However, because treatment was not standardized we cannot associate changes in plasma zonulin levels with specific interventions. In addition, the measurement of plasma zonulin levels was done before delineation of the steroid response pattern which might have led to misclassification of this clinical feature. Based on clinical practice in pediatric nephrology, most children are treated with a variety of immunosuppressive medications prior to a kidney biopsy, which was a requirement for enrollment in NEPTUNE. In this cohort, 48% of children were on immunosuppressive drugs at entry into NEPTUNE. Thus, it is unlikely that analysis of baseline samples at entry into NEPTUNE would add more information. However, this question can be readdressed in the ongoing CNEPTUNE cohort study in which pediatric patients are enrolled within 30 days of the onset of nephrotic syndrome. Treatment was not standardized and we did not collect serial samples to assess changes in plasma zonulin concentration over time or response to therapeutic interventions. However, the comparable zonulin levels in the patient subgroups on different treatment regimens suggest that the abnormal zonulin levels are intrinsic to the glomerular disease process. The NEPTUNE samples were not analyzed concurrently with a group of healthy controls and comparison was made with published values for plasma concentration in healthy subjects. However, in a follow-up validation study, we confirmed that plasma zonulin levels were higher in an independent cohort of children with NS compared to age-matched healthy controls from the CPROBE study with single batch analysis of all samples.

Zonulin is a relatively large molecule (molecular weight 47 kD) and it is not freely filtered in healthy children. The precise pathophysiological cause for the increased plasma zonulin levels in children with nephrotic syndrome (altered renal handling, synthesis, or extra-renal clearance) requires further study.

Absent a straightforward correlation between plasma zonulin levels and proteinuria, could this molecule and exposure to gluten still be a contributing factor in the pathogenesis childhood NS? In a recent study by Bouziat et al. (31), it was noted that at most 2–5% of patients with HLA susceptibility alleles who are exposed to gliadin develop celiac disease. To address this quandary, in an experimental model of celiac disease, the investigators demonstrated that exposure to reoviruses alters the immune system toward a Th1 phenotype and promotes the production of anti-gliadin antibodies and gastrointestinal inflammation. Patients with celiac disease have higher titers of antibodies to reovirus than controls that trend downward when they are placed on a gluten-free diet. Many studies have explored the role of viral infections in the pathogenesis of childhood NS. In light of the linkage between a gluten-free diet and NS disease activity, further investigation is warranted to test the hypothesis that reovirus infection or other components of the microbiome may be a second hit that modulates the immune system and triggers nephrotic syndrome in select patients with high zonulin levels. Additional clinical trials are needed to assess the potential efficacy of a gluten-free diet in the management of children with NS and to clarify the mechanism of action for this intervention.

## Conclusion

The plasma zonulin level is elevated in childhood nephrotic syndrome but it is not an indicator of disease activity. A pilot clinical trial is underway to determine if the plasma zonulin level can be used as a biomarker of a favorable response to a gluten-free diet.

## Ethics Statement

This study utilized deidentified specimens collected as part of the NEPTUNE protocol that was approved at all participating. No new specimens were collected as part of this NEPTUNE-approved ancillary study.

## Author Contributions

HT organized the study, analyzed the data, and wrote the manuscript. DG organized the study and wrote the manuscript. KL organized the study. JT analyzed the data and wrote the manuscript. CF organized the study. DM analyzed the data. SV organized the study. DA analyzed the data. JG analyzed the data and wrote the manuscript.

### Conflict of Interest Statement

The authors declare that the research was conducted in the absence of any commercial or financial relationships that could be construed as a potential conflict of interest.

## Members of the Nephrotic Syndrome Study Network (NEPTUNE)

### NEPTUNE Enrolling Centers

*Case Western Reserve University, Cleveland, OH*: J Sedor^*^, K Dell^**^, M Schachere^#^

*Children's Hospital, Los Angeles, CA*: K Lemley^*^, L Whitted^#^

*Children's Mercy Hospital, Kansas City, MO*: T Srivastava^*^, C Haney^#^

*Cohen Children's Hospital, New Hyde Park, NY:* C Sethna^*^, K Grammatikopoulos^#^

*Columbia University, New York, NY:* G Appel^*^, M Toledo^#^

*Emory University, Atlanta, GA:* L Greenbaum^*^, C Wang^**^, B Lee^#^

*Harbor-University of California Los Angeles Medical Center:* S Adler^*^, C Nast^*^, J La Page^#^

*John H. Stroger Jr. Hospital of Cook County, Chicago, IL:* A Athavale^*^, M Itteera^#^

*Johns Hopkins Medicine, Baltimore, MD:* A Neu^*^, S Boynton^#^

*Mayo Clinic, Rochester, MN:* F Fervenza^*^, M Hogan^**^, J Lieske^*^, V Chernitskiy^#^

*Montefiore Medical Center, Bronx, NY:* F Kaskel^*^, N Kumar^*^, P Flynn^#^

*NIDDK Intramural, Bethesda MD:* J Kopp^*^, E Castro-Rubio^#^, E Brede^#^

*New York University Medical Center, New York, NY:* H Trachtman^*^, O Zhdanova^**^, F Modersitzki^#^, S Vento^#^

*Stanford University, Stanford, CA:* R Lafayette^*^, K Mehta^#^

*Temple University, Philadelphia, PA:* C Gadegbeku^*^, D Johnstone^**^, Z Pfeffer^#^

*University Health Network Toronto:* D Cattran^*^, M Hladunewich^**^, H Reich^**^, P Ling^#^, M Romano^#^

*University of Miami, Miami, FL:* A Fornoni^*^, L Barisoni^*^, C Bidot^#^

*University of Michigan, Ann Arbor, MI:* M Kretzler^*^, D Gipson^*^, A Williams^#^, R Pitter^#^

*University of North Carolina, Chapel Hill, NC:* P Nachman^*^, K Gibson^*^, S Grubbs^#^, Anne Froment^#^

*University of Pennsylvania, Philadelphia, PA:* L Holzman^*^, K Meyers^**^, K Kallem^#^, FJ Cerecino^#^

*University of Texas Southwestern, Dallas, TX:* K Sambandam^*^, E Brown^**^, N Johnson^#^

*University of Washington, Seattle, WA:* A Jefferson^*^, S Hingorani^**^, K Tuttle^**^§, K Klepach^#^, S Dismuke^#^, A Cooper^#§^

*Wake Forest University, Winston-Salem, NC:* B Freedman^*^, JJ Lin^**^, M Spainhour^#^, S Gray^#^

*Data Analysis and Coordinating Center*: M Kretzler, L Barisoni, C Gadegbeku, B Gillespie, D Gipson, B Gizinski, L Holzman, L Mariani, M Sampson, P Song, J Troost, J Zee, E Herreshoff, C Kincaid, C Lienczewski, T Mainieri, A Williams

*Digital Pathology Committee*: Carmen Avila-Casado (UHN-Toronto), Serena Bagnasco (Johns Hopkins), Joseph Gaut (Washington U), Stephen Hewitt (National Cancer Institute), Jeff Hodgin (University of Michigan), Kevin Lemley (Children's Hospital LA), Laura Mariani (University of Michigan), Matthew Palmer (U Penn), Avi Rosenberg (NIDDK), Virginie Royal (Montreal), David Thomas (University of Miami), Jarcy Zee (Arbor Research) Co-Chairs: Laura Barisoni (Duke University), and Cynthia Nast (Cedar Sinai)

*National Institute of Diabetes and Digestive and Kidney Diseases (NIDDK) Program Office:* K Abbott, C Roy

*The National Center for Advancing Translational Sciences (NCATS) Program Office:* T Urv, PJ Brooks

^*^Principal Investigator; ^**^Co-investigator^; #^Study Coordinator

Cedars-Sinai Medical Center, Los Angeles, CA

^§^Providence Medical Research Center, Spokane, WA.
